# Autoinducer-2 Facilitates *Pseudomonas aeruginosa* PAO1 Pathogenicity *in Vitro* and *in Vivo*

**DOI:** 10.3389/fmicb.2017.01944

**Published:** 2017-10-17

**Authors:** Hongdong Li, Xingyuan Li, Chao Song, Yunhui Zhang, Zhengli Wang, Zhenqiu Liu, Hong Wei, Jialin Yu

**Affiliations:** ^1^Department of Neonatology, Children’s Hospital of Chongqing Medical University, Chongqing, China; ^2^Ministry of Education Key Laboratory of Child Development and Disorders, Chongqing, China; ^3^China International Science and Technology Cooperation Base of Child Development and Critical Disorders, Chongqing, China; ^4^Chongqing Key Laboratory of Child Infection and Immunity, Chongqing, China; ^5^Department of Pharmacy, Chongqing Red Cross Hospital, Chongqing, China; ^6^Shenzhen University General Hospital, Shenzhen, China

**Keywords:** *Pseudomonas aeruginosa*, autoinducer-2, biofilm, infection, virulence

## Abstract

Bacterial communication systems, such as quorum sensing (QS), have provided new insights of alternative approaches in antimicrobial treatment. We recently reported that one QS signal, named as autoinducer-2 (AI-2), can affect the behaviors of *Pseudomonas aeruginosa* PAO1 in a dose-dependent manner. In this study, we aimed to investigate the effects of AI-2 on *P. aeruginosa* PAO1 biofilm formation and virulence factors production *in vitro*, and *in vivo* using a pulmonary infection mouse model. Exogenous AI-2 resulted in increased biofilms architecture, the number of viable cells, and the yield of pyocyanin and elastase virulence factors in wild type *P. aeruginosa* PAO1. However, no such effect was observed in *P. aeruginosa lasR rhlR* mutant strain. *In vivo*, the use of AI-2 significantly increased the mortality, lung bacterial count and histological lung damage of mice with acute *P. aeruginosa* PAO1 infection. Our data suggest that AI-2 promotes the formation of *P. aeruginosa* PAO1 biofilms and the production of virulence factors by interfering with *P. aeruginosa* QS systems, resulting in decreased host survival. AI-2 may be a therapeutic target for the clinical treatment of a co-infection of *P. aeruginosa* and AI-2 producing bacteria.

## Introduction

*Pseudomonas aeruginosa* is a versatile pathogenic bacterium, which is relevant to various acute and chronic infections in immunocompromised patients, such as those with cystic fibrosis, third-degree burns, and implanted medical devices (Mathee et al., 2008; Silby et al., 2011; Gellatly and Hancock, 2013). *P. aeruginosa* infection is difficult to eradicate due to its various virulence factors including pyocyanin, rhamnolipids, elastase, exotoxin A, phospholipase C (Ben Haj et al., 2011). In particular, the formation of biofilms leads to high morbidity and mortality among infected patients because biofilms are more resistant to chemical attacks and human immune responses than planktonic bacteria (Costerton et al., 1999; Ciofu et al., 2001; Singh et al., 2002).

When bacteria grow as biofilms, they are coordinately controlled by quorum sensing (QS), a process of cell-to-cell communication that modulates various behaviors in a population density-dependent manner (Miller and Bassler, 2001). As the number of QS signaling molecules accumulate to a threshold, the QS system could be activated by the identification of specific receptors (Miller and Bassler, 2001). The QS signaling molecules mainly contain two parts, oligopeptides are commonly secreted by Gram-positive bacteria, while *N*-acyl homoserine lactones are commonly secreted by Gram-negative bacteria (Miller and Bassler, 2001). Acyl homoserine lactone (AHL)-based QS systems in *P. aeruginosa* mainly involves the *las* and *rhl* systems, which encodes two specific signal molecules named *N*-(3-oxododecanoyl)-L-homoserine lactone (3-oxo-C12-HSL) and *N*-butanoyl-L-homoserine lactone (C4-HSL), respectively (Pearson et al., 1994; Ochsner and Reiser, 1995; Miller and Bassler, 2001). In addition, researchers found that *P. aeruginosa* also employs the Pseudomonas quinolone signal (PQS) system, which can control the *rhl* system activation (Diggle et al., 2003).

AI-2, named as a universal language among the communication between bacteria, could coordinate both intra- and interspecies communication (McNab et al., 2003). It is encoded by the LuxS gene, and is the primary QS molecule produced by many Gram-positive and Gram-negative bacteria (Cho et al., 2016). It could be produced by diverse genera (almost 50) and foster inter-species communication (Hardie and Heurlier, 2008). However, *P. aeruginosa* has no ability to produce AI-2, but it can sense the molecule and then affect its function (Li et al., 2015a). Previously, we and other researchers found that AI-2, as well as AI-2 producing *Streptococcus mitis*, could affect the behaviors of *P. aeruginosa in vitro* (Duan et al., 2003; Li et al., 2015a; Wang et al., 2016). Additionally, Roy et al. (2013) found that AI-2 analogs can reduce the virulence production of *P. aeruginosa in vitro*. Unfortunately, the effect of AI-2 on *P. aeruginosa* virulence *in vivo* remains unraveled. Since effects obtained in *in vitro* model systems cannot always be repeated under *in vivo* conditions (Brackman et al., 2011), it was unclear whether AI-2 could also influence *P. aeruginosa* infections *in vivo*. Furthermore, there is a lack of direct evidence to illustrate the mechanisms of AI-2.

In the present study, we aimed to investigate the effects of AI-2 on *P. aeruginosa* biofilm formation and virulence factors production *in vitro*, as well as *in vivo* using a pulmonary infection mouse model, and to explore the roles of AI-2 in *P. aeruginosa* infections. This work may provide important insights to combat *P. aeruginosa* infections.

## Materials and Methods

### *In Vitro* Experiments

#### Bacterial Strains and Culture Conditions

*Pseudomonas aeruginosa* PAO1 (ATCC 27853) and its double mutant *P. aeruginosa lasR rhlR* (Wu et al., 2001; Feng et al., 2016) were as gifts provided by Professor Li Shen (Institute of Molecular Cell and Biology, New Orleans, LA, United States) and Professor Zhijun Song (Faculty of Health and Medical Sciences, University of Copenhagen, Copenhagen, Denmark), respectively. Both bacteria were incubated at 37°C with shaking (200 rpm). Chemically synthesized AI-2 precursor DPD [(*S*)-4, 5-dihydroxy-2, 3-pentanedione] was obtained from Omm Scientific company (Dallas, TX, United States). The AI-2 used in this study was chosen as a biological relevant concentration, because 10 nM AI-2 resulted in the greatest response in virulence factor production and biofilm formation (Li et al., 2015a). Furthermore, AI-2 producing *S. mitis* and *Klebsiella pneumoniae* were the most frequent microbes in the biofilms on the surface of neonatal endotracheal tubes extubated from mechanically ventilated newborns, and the AI-2 concentration secreted by these AI-2 producing bacteria in the biofilms on the surface of neonatal endotracheal tubes was about 10–50 nM (Li et al., 2015b; Wang et al., 2016; Pan et al., 2017).

#### Biofilm Formation Assays

The biofilm formation assay was performed under a static environment in 96-well polystyrene microtiter plates as previously described (Sarkar et al., 2014). In brief, an optical density at 600 nm (OD_600_) of 0.05 diluted cultures with 10 nM AI-2 were added to 96-well microtiter plates (Costar, United States), and 8 replicates were added in each group. After 24 h at 37°C without shaking, the plates were washed by phosphate-buffered saline (PBS) at least three times. Then, the plates were dried and stained with 0.1% crystal violet. The bacteria-bound crystal violet was dissolved in 200 μL 95% ethanol, and the absorbance at 570 nm was determined. All experiments were performed three times independently (*n* = 24).

#### Confocal Laser Scanning Microscopy (CLSM) and Viability Counts

*Pseudomonas aeruginosa* PAO1 and *P. aeruginosa lasR rhlR* mutant cells were inoculated in LB broth at a standard concentration (A600 = 0.05), then the dilutions were added to glass coverslips (Costar, United States) which were laid in a 24-well plate (3 replicates were added in each group), followed by growth for 48 h without shaking at 37°C. Coverslips were then washed three times with PBS and stained with SYTO9/propidium iodide according to the manufacturer’s instructions of the L13152 LIVE/DEAD BacLight bacterial viability kit (Invitrogen Molecular Probes, United States). Coverslips were visualized by a Nikon A1R laser confocal microscope (Nikon, Tokyo, Japan). Live bacteria were stained green (excitation 488 nm, emission 515/30 nm) while dead bacteria were stained red (excitation 568 nm, emission 600/50 nm). In parallel experiments, samples were also prepared for viability counts at 24 and 48 h, respectively.

#### Virulence Factor Assays

For the pyocyanin assay, 3 mL supernatant from a 24 h culture in pyocyanin production broth (PPB; 2% proteose peptone [Oxoid, UK], 1% K_2_SO_4_, 0.3% MgCl_2_⋅6H_2_O) was extracted by chloroform, and further re-extracted with 0.2 M HCl, a red layer was obtained. The optical density was determined at 520 nm (Kong et al., 2005). Elastase activity in the supernatant was processed using the elastin-Congo red (ECR) assay as previously described (Yang et al., 2012). Briefly, filtered supernatant from a 6 h culture in peptone tryptic soy broth (PTSB; 5% peptone, 0.1% tryptic soy broth) was added to a new tube containing 10 mg of ECR (Sigma, United States), 900 μL 10 mM Tris HCl (pH 7.5), and 1 mM CaCl_2_. The tubes were incubated for 4 h at 37°C with agitation. At last, unreacted substrate was removed by centrifugation. The optical density was determined at 495 nm. Five replicates were in each group and all experiments were performed three times independently.

### *In Vivo* Experiments

#### Animals

All experiments were performed in accordance with relevant guidelines and regulations, and the animal experimental protocol was approved by the Animal Care and Use Committee at the Chongqing Medical University. Male C57BL/6 mice aged 6–8 weeks were purchased from Chongqing Medical University (Chongqing, China). All mice were housed in individually filtered cages and received unlimited sterile food and water in the Laboratory Animal Center at the Children’s Hospital of Chongqing Medical University.

#### Mouse Model of *P. aeruginosa* Acute Lung Infection

An experimental model of acute lung infection was established following the method of Song et al. with some modifications (Song et al., 2014). The concentration of *P. aeruginosa* cultures were adjusted to 0.25(A_600_). Inoculated bacterial counts were confirmed by plating the suspensions on LB plates (Sangon, Shanghai, China). Mice were infected with an intratracheal instillation of 0.05ml of bacterial suspension (1 × 10^7^ colony-forming units/mouse) under anesthesia with pentobarbital sodium. As control, intratracheal instillation was performed with 0.05 ml PBS or 10 nM AI-2 in the same manner. Thus, there were four groups used in the study: (i) infection control group: infection was given with PAO1 only (P group); (ii) AI-2 treated group: infection was given with PAO1 and AI-2 (P+AI-2 group); (iii) AI-2 control group: treatment was given with AI-2 only (AI-2 group); (iv) PBS control group: treatment was given with PBS only (PBS group). For survival studies, 12 mice in each group were observed once daily for 7 days post infection. Other mice (a total of 60 mice with 5 mice in each group for each experiment) were sacrificed by cervical dislocation after 24 h of the infection, and the lungs and bronchoalveolar lavage fluid (BALF) were collected for further analysis.“(see below).”

#### Bacteriological and Histopathological Examination

For bacteriological analysis, the lungs were suspended in 1 ml of cold sterile PBS and homogenized using a homogenizer (Changzheng Co., Chongqing, China) at 4°C. The specimen was serially diluted and plated on *P. aeruginosa* selective agar to measure the CFU per lung, followed by incubation at 37°C for 16–20 h. For histopathological analysis, lung tissues were fixed in 10% neutral buffered formalin for 48 h, and the paraffin-embedded sections, cut into 5 μm sections, were stained with hematoxylin-eosin (H&E; Sigma). Light microscopy (Nikon eclipse 55i, Japan) was used to obtain the images.

#### Analysis of BALF

Mice were anesthetized with pentobarbital sodium at 24 h after *P. aeruginosa* infection, an appropriate plastic intravenous catheter was inserted into the trachea, BALF was collected by three times washes with ice-cold PBS, and total cell numbers in BALF were measured. Cell-free BALF supernatants were collected by centrifugation (3000 g for 5 min at 4°C) and stored at -80°C prior to cytokine profile characterization. The pro-inflammatory cytokines tumor necrosis factor alpha (TNF-α), IL-6 and the anti-inflammatory cytokine IL-10 concentration in the supernatant fluid were determined by using mouse cytokine enzyme-linked immunosorbent assay kits (Sizhengbai, Beijing, China) according to the manufacturer’s instructions.

### Statistical Analysis

All data from the study are expressed as means ± SD. Analysis of variance (ANOVA) was used to determine the level of significant differences between all groups and Tukey’s honest significant difference (HSD) for pairwise comparison. Survival analysis was performed using the log-rank (Mantel-Cox) test. *P* < 0.05 was considered to be statistically significant.

## Results

### *In Vitro* Experiments

#### AI-2 Increased *P. aeruginosa* PAO1 Biofilm Formation

**FIGURE 1:**
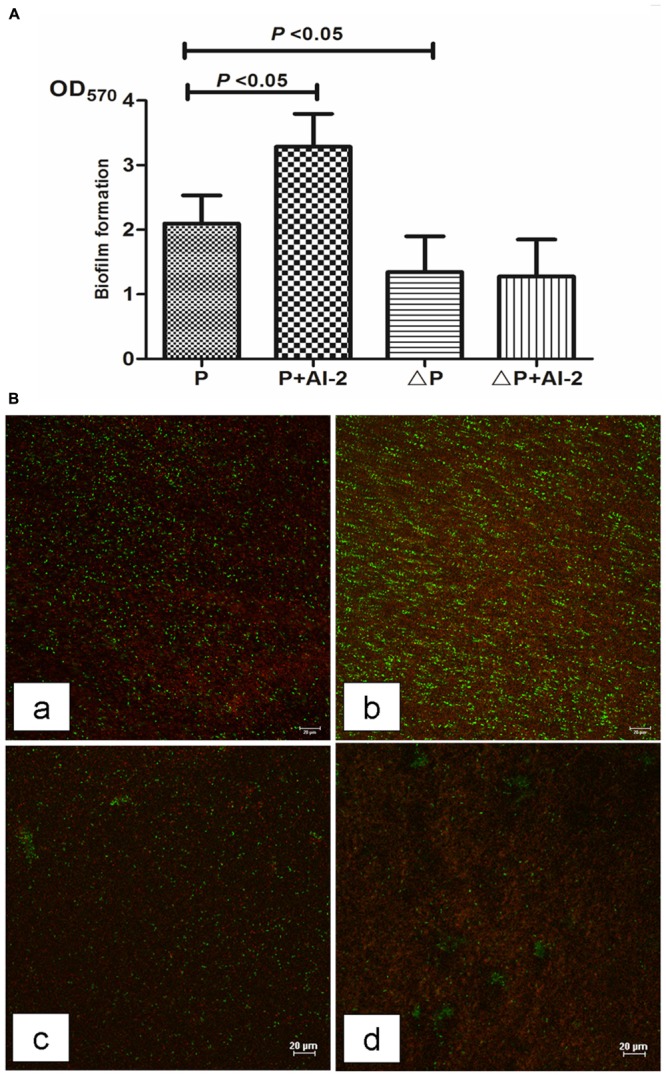
Effects of AI-2 on *P. aeruginosa* PAO1 biofilm formation. P, PAO1 group; P+AI-2, PAO1+AI-2 group; ΔP, *P. aeruginosa lasR rhlR* mutant group; ΔP+AI-2, *P. aeruginosa lasR rhlR* mutant+AI-2 group. **(A)** Biofilm formation was assessed by crystal violet. Error bars represent SD and all experiments were performed in three times independently (*n* = 24); **(B)** Representative confocal laser scanning micrographs of biofilms in four groups. Compared to the PAO1 control group (a), a dense and compact biofilm and more live bacteria were observed under CLSM in P+AI-2 group (b) and a loosend biofilm structure and fewer viable cells were visualized in the ΔP+AI-2 group (c) and ΔP group (d). Red, non-viable cells; green, viable cells. scale bars = 20 μm.

First, we monitored wild type *P. aeruginosa* PAO1 biofim formation with and without 10 nM AI-2. Crystal violet assay revealed that AI-2 added culture (P+AI-2 group) resulted in a significant increase in the total biomass by 61.4% as compared to PAO1 control group (P group) (**Figure 1A**). Notably, consistent with biomass results, Two-dimensional (2D) imaging revealed a more dense biofilm architecture under CLSM in the P+AI-2 group, and more viable cells were visualized (**Figure 1B**). In addition, the number of viable bacteria in the P+AI-2 group was significantly higher than that in the P group (**Table 1**), the amount of bacteria released from the biofilms at 24 h in the P+AI-2 group increased by 80.6% as compared to P group [(5.6 ± 0.5) × 10^7^ CFU/cm^2^ vs. (3.1 ± 0.4) × 10^7^ CFU/cm^2^, *F*(3,36) = 23.25, *P* = 0.03]. In addition, we used the same method to assess the effect of AI-2 on *P. aeruginosa lasR rhlR* mutant strain. The results showed that biofilm formation of the mutant strain was impaired when compared to the wild type PAO1. Furthermore, the addition of AI-2 to *P. aeruginosa lasR rhlR* mutant cultures (ΔP+AI-2 group) resulted in no significant changes as compared to *P. aeruginosa lasR rhlR* mutant group (ΔP group) (**Figure 1** and **Table 1**).

**Table 1:** Viable bacterial counts recovered from biofilms formed on coverslips.

Groups	CFU/cm^2^ biofilm	
	24 h	48h
P	(3.1 ± 0.4) × 10^7^	(1.2 ± 0.3) × 10^8^
P+AI-2	(5.6 ± 0.5) × 10^7∗^	(3.2 ± 0.6) × 10^8 ▲^
ΔP	(7.5 ± 0.38) × 10^6∗^	(5.8 ± 0.5) × 10^7 ▲^
ΔP+AI-2	(9.2 ± 0.4) × 10^6^	(6.6 ± 0.43) × 10^7^

*P, PAO1 group; P+AI-2, PAO1+AI-2 group; ΔP, *P. aeruginosa lasR rhlR* mutant group; ΔP+AI-2, *P. aeruginosa lasR rhlR* mutant+AI-2 group. Asterisks denote a statistically significant difference from the PAO1 control group at 24 h (*P* < 0.05). Triangles denote a statistically significant difference from the PAO1 group at 48h (*P* < 0.05).*

#### AI-2 Increased *P. aeruginosa* PAO1 Virulence Factors Production

**FIGURE 2:**
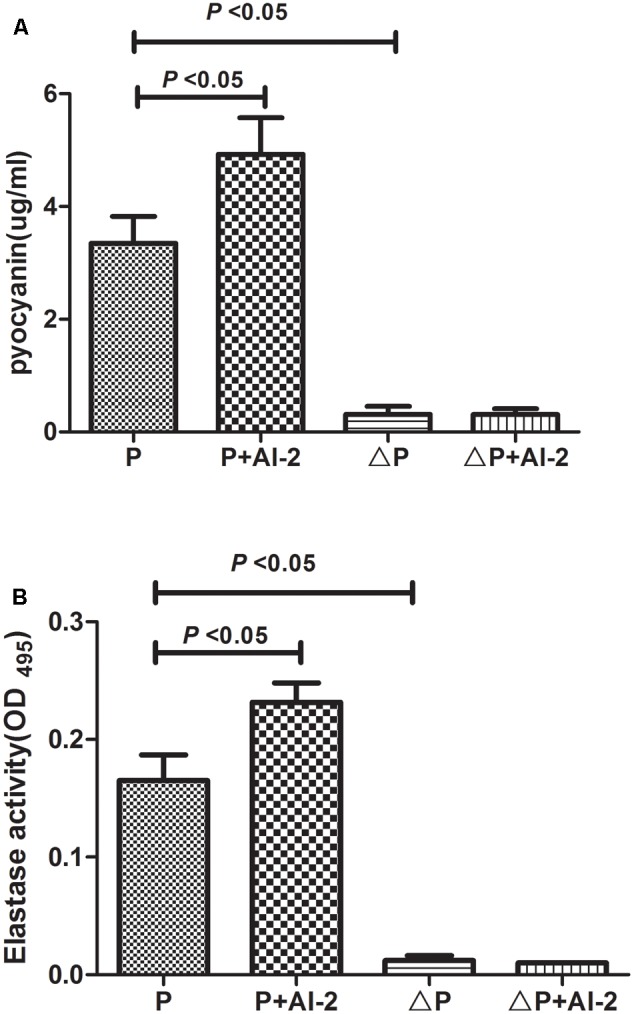
Effects of AI-2 on the production of virulence factors of *P. aeruginosa* PAO1. P, PAO1 group; P+AI-2, PAO1+AI-2 group; triangleP, *P. aeruginosa lasR rhlR* mutant group; triangleP+AI-2, *P. aeruginosa lasR rhlR* mutant+AI-2 group. **(A)** Relative productions of virulence factors of pyocyanin. **(B)** Elastase activity.

Pyocyanin and elastase, controlled by the *P. aeruginosa* QS system, are two very important virulence factors (Ben Haj et al., 2011). Whether AI-2 could promote the production of these two virulence factors was determined. As shown in **Figure 2**, the activity of both pyocyanin and elastase were significantly increased in the P+AI-2 group [*F*(3,57) = 99.03, *P* = 0.004 and *F*(3,57) = 162.33, *P* = 0.001, respectively] while there was no such effect in ΔP+AI-2 group.

### *In Vivo* Experiments

#### AI-2 Increased Mortality Rates in Mouse Models of Lung Infection

**FIGURE 3:**
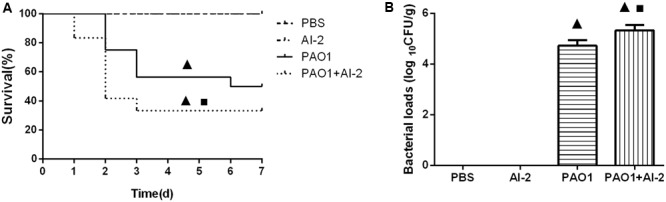
Analysis of survival data and bacterial loads after infection. The animals were treated with PBS, AI-2, *P. aeruginosa* PAO1 (P) and AI-2+*P. aeruginosa* PAO1 (P+AI-2), respectively. Triangles denote a statistically significant difference from the PAO1 group (*P* < 0.05), asterisks denote a statistically significant difference from the PBS group (*P* < 0.05). **(A)** Survival was monitored for 7 days (*n* = 12 mice per group); **(B)** Bacterial loads in the lungs after infection for 24 h.

To evaluate the effects of AI-2 on mouse models *in vivo*, we first determined the mortality rates of the animals which were co-infected with AI-2 or not. The mortality rate was 0 (0/12) in the PBS or AI-2 group (**Figure 3A**), indicating that AI-2 itself has no effect on animals. The spontaneous mortality rate was 50% (6/12) in the PAO1 control group (P). However, when the mice were co-infected with *P. aeruginosa* PAO1 and AI-2, the mortality rate was significantly increased to 66.7% (8/12) (*P* < 0.05).

#### AI-2 Increased *P. aeruginosa* Local Infection in the Lungs

We further collected the lungs which were infected 24 h post infection for bacteriological analysis. There was no *P. aeruginosa* colonizing in the lungs in AI-2 group and PBS group. However, the bacterial count in the lungs of P+AI-2 group (5.2 ± 0.15 Log CFU/g) was significantly more than that in P group (4.4 ± 0.1 Log CFU/g) [F(3, 17) = 102.5, *P* = 0.004] (**Figure 3B**).

#### AI-2 Aggravated *P. aeruginosa* -Induced Lung Injury and Inflammation

**FIGURE 4:**
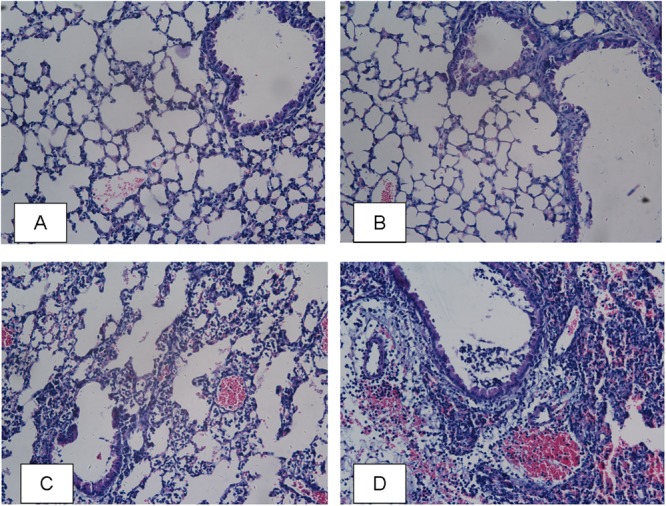
Lung histological examination after infection. Sections of lungs stained with hematoxylin-eosin at 24 h post infection are shown (magnifications, × 400). **(A)** PBS group; **(B)** AI-2 group; **(C)** *P. aeruginosa* PAO1 group; **(D)** *P. aeruginosa* PAO1+AI-2 group.

**FIGURE 5:**
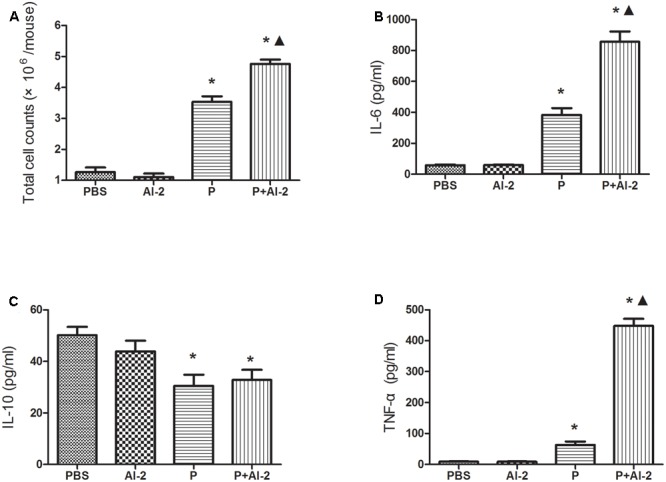
Effects of AI-2 on BALF cytokine levels. BALF was collected at 24 h post infection. Each sample was analyzed by an ELISA kit. The mice were infected with PBS, AI-2, *P. aeruginosa* PAO1 (P) and AI-2 + *P. aeruginosa* PAO1 (P+AI-2), respectively. Total cells **(A)**, IL-6 **(B)**, IL-10 **(C)** and TNF-α **(D)** levels present in the BALF of the respective treatment groups are shown. Triangles denote a statistically significant difference from the PAO1 group (*P* < 0.05), asterisks denote a statistically significant difference from the PBS group (*P* < 0.05).

To evaluate the pro-inflammatory effects of AI-2, lung tissues were harvested post infection. The P+AI-2 group exhibited a higher bacterial loads and more serious pathological changes. In P+AI-2 group mice, more leukocytes were found to infiltrate into the peribronchiolar and perivascular connective tissues, and the neutrophil cells were the primary infiltrating cell types (**Figure 4**). Moreover, to examine whether AI-2 could aggravate the inflammatory cell infiltration into the lungs following PAO1 infection, the total cells present in the BALF were counted. As shown in **Figure 5A**, the median total number of BALF cells in P+AI-2 group was 4.75 × 10^6^ cells/mouse, as compared to 3.5 × 10^6^ cells/mouse in P group [*F*(3,17) = 153.25, *P* = 0.002]. To further assess the effects of AI-2 on BALF cytokine profile changes, ELISA-based assays were used. The levels of cytokines (TNF-α, IL-6) in the BALF were significantly higher in the P+AI-2 group than those in the P group [*F*(3,17) = 91.03, *P* = 0.005 and *F*(3,17) = 162.33, *P* = 0.001, respectively] (**Figures 5B,D**). IL-10 levels tended to be higher in the P+AI-2 group than in the P group, although the increase was not statistically significant (**Figure 5C**). Therefore, these results suggest that AI-2 could aggravate lung injury and inflammation by increasing bacterial counts.

## Discussion

In this study, both *in vitro* and *in vivo* results demonstrated that AI-2 could influence *P. aeruginosa* PAO1 biofilm formation and virulence factor production by interfering with *P. aeruginosa* QS systems, leading to an increased lung infection and mortality rate. *P. aeruginosa* is a common bacterium that can cause various serious infections in human body (Brennan and Geddes, 2002). Recently, we found it is also one of the most prevalent bacteria on endotracheal tubes (ETT) samples of neonates who were under mechanical ventilation (Li et al., 2015b). One interesting phenomenon is that AI-2 producers *Klebsiella* spp. and *Streptococcus* spp. often coexist with *P. aeruginosa* on the same ETT samples (Li et al., 2015b). Biofilms harbor a complex microbiological community, which means a specific environment on the surface of neonatal ETTs. As AI-2 is a universal signal for intra- and interspecies communication, AI-2 signaling pathway is a potential target to inhibit biofilms infections (Jang et al., 2013; Roy et al., 2013).

The role of AI-2 as a general bacterial signaling molecule affecting biofilm formation *in vitro* has been widely studied. AI-2 inhibits biofilm formation in *Bacillus cereus* (Auger et al., 2006), *Candida albicans* (Bachtiar et al., 2014), and *Eikenella corrodens* (Azakami et al., 2006), and it promotes biofilm formation in *Escherichia coli* (Gonzalez Barrios et al., 2006), *Streptococcus mutans* (Yoshida et al., 2005), and multispecies biofilms between the two oral bacteria *Streptococcus gordonii* and *Porphyromonas gingivalis* (McNab et al., 2003). However, to the best of our knowledge, the addition of exogenous AI-2 to bacterial acute pneumonia is seldom investigated, and this is the first study aimed at revealing the AI-2 effects on *P. aeruginosa* infections *in vivo*.

Based on the positive interaction observed between AI-2 and *P. aeruginosa* in *in vitro* studies, we further established a pulmonary infection mouse model to investigate the effects of AI-2 on *P. aeruginosa* infections *in vivo*. Lower rates of survival and more serious infections were observed in the P+AI-2 group than in the P group as a result of higher bacterial loads, more serious pathological changes, and more inflammatory cells infiltrating in the BALF. Similarly, Duan et al. (2003) reported that AI-2 producing bacteria could aggravate the lung injury when co-infected with *P. aeruginosa*. This phenomenon may be attributed to the higher secretion of elastase and pyocyanin, which was regulated by QS genes and virulence factor genes. We previously found that AI-2 could enhance the expression of the *P. aeruginosa* PAO1 QS genes and virulence factor genes, including *lasI, lasR, rhlI, rhlR, lasB, lasA*, and *phzH* (Li et al., 2015a). Pyocyanin in the lungs of mice has been shown to cause a number of pathological changes including airway fibrosis, goblet cell hyperplasia and metaplasia, and destruction of the alveolar airspaces (Caldwell et al., 2009). Furthermore, Yilmaz et al. (2015) found that pyocyanin could contribute to the virulence of *Porphyromonas gingivalis* and disease severity. Besides, elastase could break down hemoglobin and lead to pulmonary hemorrhage (Cosgrove et al., 2011). In this study, the concentration of IL-10 in the BALF had no significant difference between P group and P+AI-2 group (*P* > 0.05), but both groups showed significantly higher concentration than in PBS control group (*P* < 0.05). IL-10 is an anti-inflammatory cytokine and a key immunoregulator during infection. High pathogen loads drive immoderate Th1 responses (Anderson et al., 2005), and in turn, promotes the development of self-limiting inflammation and adaptive immune responses. IL-10-producing T cells could dampen the Th1 response (Belkaid et al., 2001; Anderson et al., 2007). These events form a positive feedback loop whereby IL-10 further inhibits antimicrobial immune responses, allowing inevitably serious infections to develop (Couper et al., 2008).

QS is known to play a key role in biofilm formation and virulence production (Miller and Bassler, 2001). Thus, it can be a suitable target for antimicrobial therapy. It is noteworthy that the *P. aeruginosa lasR rhlR* mutant has weaker biofilm formation and reduced virulence factor production as compared with PAO1 (**Figures 1, 2**). Meanwhile, *P. aeruginosa* QS mutations are very common in isolates from cystic fibrosis patients with chronic infection (Jiricny et al., 2014). Currently, chemicals that interfere with QS systems are being studied. Brackman et al. (2011) found that quorum-sensing inhibitors (QSI) increased the susceptibility of bacterial biofilms to antibiotics both *in vitro and in vivo*. Christensen et al. found that the QSIs furanone C-30, ajoene or horseradish juice extract could have a synergistic antimicrobial efficacy when used in combination with tobramycin (Christensen et al., 2012). Furthermore, we have demonstrated that D-ribose (an AI-2 analog) could reverse the effects produced by two common pathogens on ETT, *S. mitis* and *P. aeruginosa* (Wang et al., 2016). However, the results from our study seemed promising, and it may be of significance to determine whether these effects can be obtained for other common pathogens on ETT (such as *Klebsiella pneumoniae*). On the other hand, we would further explore if AI-2 could enhance the biofilm formation of *P. aeruginosa* in *in vivo* models and the cytotoxicity of AI-2 as higher concentrations might be toxic. Hoffmann et al. (Hoffmann et al., 2005) found that biofilms were established in a mouse model of chronic *P. aeruginosa* lung infection mimicking cystic fibrosis. In future studies, it will therefore be important to clarify these mechanisms.

## Conclusion

This study demonstrated that AI-2 increased *P. aeruginosa* PAO1 pathogenicity both *in vitro* and *in vivo*, and AI-2 increased the mortality rate and aggravated the lung infection in a mouse model of acute *P. aeruginosa* pneumonia by secreting more virulence factors. Our results support the significance of AI-2 in the regulation of *P. aeruginosa* PAO1. AI-2 may provide novel means of combating clinical co-infection of *P. aeruginosa* and AI-2 producer bacteria by interfering with bacterial signaling.

## Author Contributions

HL and JY conceived and designed this study. HL, CS, ZW, and YZ performed the experiments. HL, XL, ZL, and HW analyzed the data and wrote the paper. HL, ZL, and HW revised the manuscripts.

## Conflict of Interest Statement

The authors declare that the research was conducted in the absence of any commercial or financial relationships that could be construed as a potential conflict of interest.
